# Significantly Lower Saliva Secretion in Females With Gastroesophageal Reflux Disease and Healthy Subjects Than in Males

**DOI:** 10.7759/cureus.39020

**Published:** 2023-05-15

**Authors:** Eri Momma, Mai Koeda, Yoshimasa Hoshikawa, Tomohide Tanabe, Shintaro Hoshino, Noriyuki Kawami, Katsuhiko Iwakiri

**Affiliations:** 1 Department of Gastroenterology, Nippon Medical School, Graduate school of Medicine, Tokyo, JPN; 2 Department of Gastroenterology, Nippon Medical School, Graduate School of Medicine, Tokyo, JPN

**Keywords:** non-erosive reflux disease, reflux esophagitis, gastroesophageal reflux disease, saliva, sex difference

## Abstract

Objective: Saliva secretion in healthy subjects is lower in females than in males. The present study investigated sex differences in saliva secretion in patients with gastroesophageal reflux disease (GERD) and healthy controls.

Methods: This case-control study included 39 (male/female: 16/23) with non-erosive reflux disease (NERD), 49 (25/24) patients with mild reflux esophagitis, 45 (23/22) with severe reflux esophagitis (A1), and 46 (24/22) healthy controls. Saliva secretion was examined as follows: before endoscopy, patients chewed sugar-free gum for three minutes, and the amount and pH of saliva before and after acid loading as an index of acid-buffering capacity were evaluated. The relationships between saliva secretion and body mass index, height, and weight were also examined.

Results: The amount of saliva secreted was significantly lower in females than in males in all four groups (NERD, mild reflux esophagitis, severe reflux esophagitis, and healthy controls). Salivary pH and acid-buffering capacity were similar in all groups. The amount of saliva secreted positively correlated with height and body weight, albeit more strongly with height.

Conclusion: A sex difference in saliva secretion exists in GERD patients, similar to healthy controls. Saliva secretion was significantly lower in female GERD patients than in male GERD patients.

## Introduction

Reflux esophagitis (RE) is characterized by the excessive exposure of the esophagus to acid [1-3]. Acid exposure times have been associated with esophageal body dysmotility [4,5] and salivation capacity [6,7]. In our previous study, we showed that the amount of stimulated saliva secretion was significantly lower in patients with proton pump inhibitor (PPI)-resistant severe RE than in PPI-responsive RE [8], and in non-erosive reflux disease (NERD) patients [9,10] or mild RE [10,11] than in healthy controls (HCs).

A previous study reported a higher incidence of collagen disease in patients with PPI-resistant severe RE than in those with PPI-responsive severe RE as well as a higher number of females among the former than the latter [8]. A comparison of saliva secretion in patients with PPI-resistant RE with or without collagen disease revealed a decrease in saliva secretion regardless of collagen disease [8], suggesting a sex difference in PPI resistance. Although stimulated saliva secretion in HCs was found to be lower in females than in males at all ages [12], few studies have investigated sex differences in saliva secretion in gastroesophageal reflux disease (GERD) patients [13]. Moreover, since females are more susceptible to NERD [1,14], sex differences in saliva secretion may be related to the etiology of GERD. Saliva secretion affects the duration of esophageal acid exposure; therefore, it is important to establish whether sex differences in saliva secretion exist in GERD patients similar to HCs.

In the present study, we investigated sex differences in saliva secretion in patients with GERD (NERD, mild RE, and severe RE) and HCs, and we also examined the relationships between saliva secretion and body mass index (BMI), height, and weight, since body size has been identified as a factor contributing to sex differences in saliva secretion [15].

## Materials and methods

We conducted this retrospective case-control analysis at Nippon Medical School Hospital from October 2018 to August 2022. RE defined as grade A or B by the Los Angeles classification was categorized as mild RE in the present study and grade C or D as severe RE. Patients without RE (grades A-D) on endoscopy who exhibited reflux symptoms (heartburn and/or regurgitation) for which acid-suppressive therapy was effective were diagnosed with NERD.

The number of samples was selected based on a re-evaluation of our previous study [8] in which sex differences in saliva secretion were examined in patients with PPI-resistant or PPI-responsive severe RE. The amount of stimulated saliva secretion in 3 minutes was less than 4.1 mL in approximately 5% of males and 75% of females. Ten subjects were needed in each group for at least a 70% difference to be detected between the groups by Fisher’s exact test with a two-sided alpha error of 0.05 and a power of 0.80. IBM SPSS Sample Power 3.0.1 (IBM, Armonk, NY, USA) was used to calculate sample powers.

Since this was a retrospective analysis, information was collected on 10 or more consecutive patients of each sex in each group (NERD, mild RE, severe RE, and HC) treated in the previous 34 months. Therefore, 39 patients with NERD (16 males and 23 females), 49 with mild RE (25 males and 24 females), 45 with severe RE (23 males and 22 females), and 46 HCs (24 males and 22 females) were enrolled in this study. Acid-suppressive therapy (PPI or a potassium-competitive acid blocker (PCAB)), which was previously shown to have no effects on saliva secretion [16,17], effectively controlled esophageal mucosal damage and/or reflux symptoms in all GERD patients. HCs, who were consecutively recruited throughout the study period, had an abnormal finding detected during a regular health check-up. Follow-up endoscopy showed no abnormalities other than atrophic gastritis or they were being followed up after the eradication of Helicobacter pylori. None of the HCs exhibited any symptoms or were receiving any medication. Regarding revised F scale scores [18] in HCs before endoscopy, symptoms were scored as ≥1 point and the total symptom score was ≤3 points.

Patients receiving drugs that have been confirmed to decrease the secretion of saliva, including antihypertensive drugs (adrenergic alpha-2 agonists and anti-adrenergic agents), antidepressants, psychotropic drugs, and anticholinergic drugs, and cigarette smokers [19] were excluded from the analysis.

All patients and HCs were subjected to a saliva secretion test and esophagogastroduodenoscopy (EGD). The amount of fasting stimulated saliva secretion on the day of endoscopy was measured at approximately 9:00 a.m. before EGD using the saliva secretion test. Patients and HCs chewed sugar-free gum (CAT 21, J. Morita Corp., Tokyo, Japan) for three minutes and the amount and pH of saliva secreted were then assessed. After loading 50 μL of 0.1 N HCl per 0.5 mL of saliva, pH was evaluated as acid-buffering capacity [20,21].

After saliva had been collected, EGD was conducted to establish whether esophageal mucosal breaks, hiatus hernia, or gastric mucosa atrophy was present. Gastric mucosa atrophy was assessed according to the Kimura-Takemoto Classification [22]; no atrophy was classified as C1. A length >2 cm between the hiatus and the lower margin of the esophageal palisade vessels was diagnosed as a hiatus hernia [23]. BMI was also measured.

Statistical analysis

Data are shown as medians (25th-75th percentiles). Differences in age, BMI, the amount of saliva secreted, salivary pH, and acid-buffering capacity were examined using the Mann-Whitney U test. Sex, the presence of gastric mucosal atrophy, and the presence of hiatus hernia were compared between the groups by Fisher’s exact test. Relationships between the amount of saliva secreted and height, body weight, and BMI were investigated using Pearson’s correlation coefficient. P < 0.05 indicated a significant difference.

Ethics approval and consent to participate

The present study was conducted according to the ethical standards of the responsible committees on human experimentation (institutional and national) and the Declaration of Helsinki 1964 and its later versions. The Ethics Committee of Nippon Medical School Hospital approved the protocol of the present study (B-2021-481). Since this was a retrospective analysis, the need for written informed consent was waived.

## Results

Patient characteristics

Patient characteristics in each group are shown in Table 1. Males were significantly older than females in the severe RE group (p=0.002). No significant differences were observed in age between males and females in the other groups examined. Furthermore, the presence of hiatus hernia and gastric atrophy did not significantly differ between males and females in any group.

**Table 1:** Clinical characteristics and demographic data of patients with non-erosive reflux disease (NERD), mild and severe reflux esophagitis (RE), and healthy control groups Gastric mucosal atrophy was classified according to the Kimura-Takemoto classification and C1 was considered to have no atrophy. BMI=Body Mass Index Statistical analyses were performed by the Mann-Whitney U test * or Fisher’s exact test**.

	NERD Group			Mild RE Group			Severe RE Group			Healthy Group		
	Male (n＝16)	Female (n＝23)	P	Male (n＝25)	Female (n＝24)	P	Male (n＝23)	Female (n＝22)	P	Male (n＝24)	Female (n＝22)	P
Age median (25-75%)	66.5 (57.5-77.0)	71.0 (59.0-78.0)	0.877*	68.5 (58.3-73.3)	69.0 (62.3-72.0)	0.726*	63.0 (50.0-74.0)	79.0 (67.5-83.0)	0.002*	58.0 (50.0-69.8)	57.0 (48.3-69.8)	0.733*
Hernia ∔/-	9/7	13/10	0.987**	18/7	17/7	>0.999**	20/3	19/3	0.953**	4/20	3/19	0.775**
Atrophy ∔/-	2/14	7/16	0.191**	4/21	3/21	0.7030**	1/22	0/22	0.323**	8/16	5/17	0.425**
BMI (25-75%)	23.8 (23.1-26.3)	21.5 (18.9-25.1)	0.051*	24.0 (22.7-25.6)	21.9 (21.0-24.6)	0.1010*	24.6 (21.8-26.9)	21.1 (19.0-24.0)	0.1156*	23.7 (21.1-25.7)	20.1 (18.5-21.8)	0.0017*
Height (cm) (25-75%)	170.0 (161.5-173.5)	156.0 (151.2-158.8)	<0.0001*	168 (1650.-173.3)	153.0 (148.5-157.0)	<0.0001*	170.0 (168.0-174.5)	153.0 (146.0-156.0)	<0.0001*	170.0 (168.5-177.0)	158.0 (156.0-162.0)	<0.0001*
Body weight (kg) (25-75%)	68.5 (63.0-73.0)	53.0 (45.3-56.5)	<0.0001*	68.0 (64.0-75.3)	53.0 (48.5-59.0)	<0.0001*	71.0 (63.3-81.5)	48.5 (43.0-55.0)	<0.0001*	68,5 (60.6-78.0)	50.0 (46.0-55.0)	<0.0001*

Sex differences in saliva secretion in the NERD group

**Figure 1:**
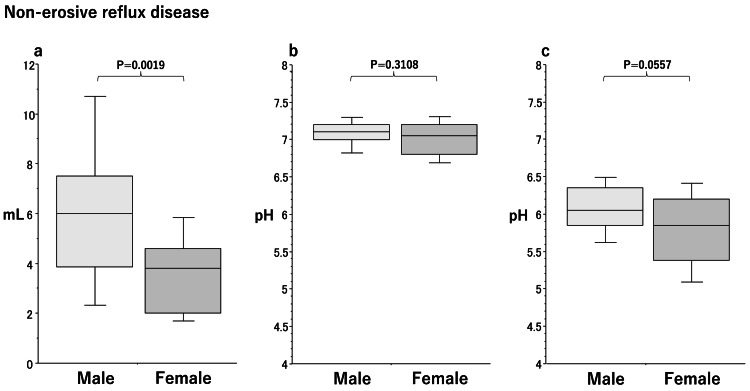
Box plots of sex differences in the amount of stimulated saliva secretion (a), salivary pH (b), and acid-buffering capacity (c) in patients with non-erosive reflux disease (NERD). Significant differences were calculated by the Mann-Whitney U test. Lower and upper whiskers indicate 10th and 90th percentiles, respectively. Box plots indicate 25th, 50th, and 75th percentiles.

The amount of saliva secreted (Figure 1a) was significantly lower (p=0.019) in females (3.8 [2.0-4.6]) than in males (6.0 [3.9-7.5]) in the NERD group. No significant differences were noted in salivary pH (male/female, 7.1 [7.0-7.2]/7.1 [6.8-7.2], p=0.3108) (Figure 1b) or acid-buffering capacity (male/female, 6.1 [5.9-6.4]/5.8 [5.3-6.2], p=0.0557) (Figure 1c) between males and females.

Sex differences in saliva secretion in the mild RE group

**Figure 2:**
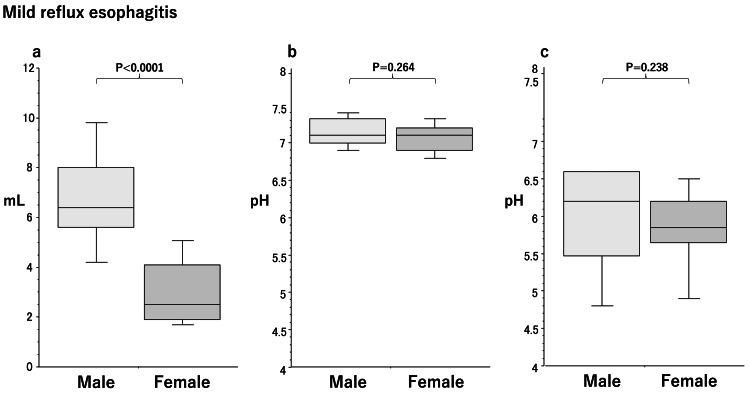
Box plots of sex differences in the amount of stimulated saliva secretion (a), salivary pH (b), and acid-buffering capacity (c) in patients with mild reflux esophagitis. Significant differences were calculated by the Mann-Whitney U test. Lower and upper whiskers indicate 10th and 90th percentiles, respectively. Box plots indicate 25th, 50th, and 75th percentiles.

The amount of saliva secreted (Figure 2a) was significantly lower (p<0.001) in females (2.5 mL/3 minutes [1.9-4.1] median [25th-75th percentiles]) than in males (6.4 [5.6-8.0]) in the mild RE group. No significant differences were observed in salivary pH (male/female, 7.1 [7.0-7.3]/7.1 [6.9-7.2], p=0.2340) (Figure 2b) or acid-buffering capacity (male/female, 6.2 [5.5-6.6]/ 5.9 [5.7-6.2], p=0.2380) (Figure 2c) between males and females.

Sex differences in saliva secretion in the severe RE group 

**Figure 3:**
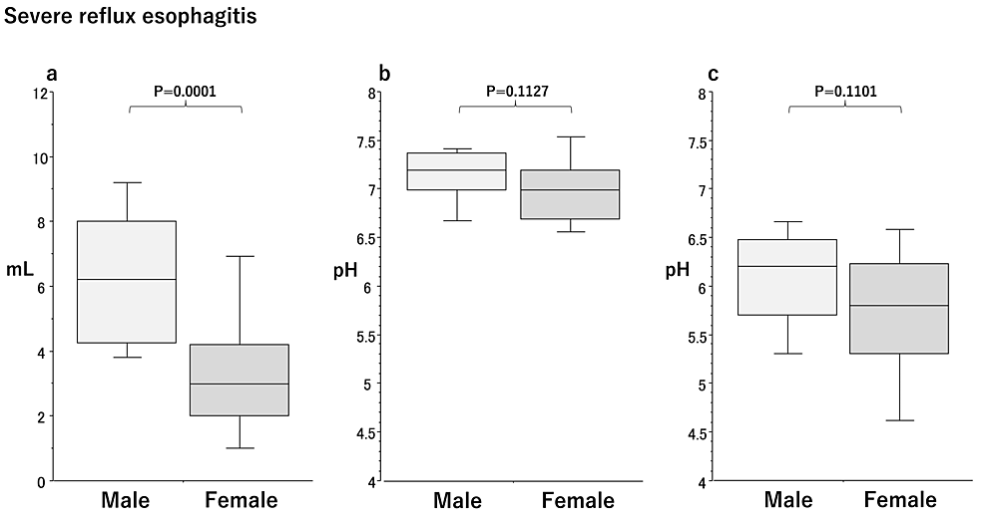
Box plots of sex differences in the amount of stimulated saliva secretion (a), salivary pH (b), and acid-buffering capacity (c) in patients with severe reflux esophagitis. Significant differences were calculated by the Mann-Whitney U test. Lower and upper whiskers indicate 10th and 90th percentiles, respectively. Box plots indicate 25th, 50th, and 75th percentiles.

The amount of saliva secreted (3.0 [2.0-4.2], p=0.0001) (Figure 3a) was significantly lower in females than in males (6.2 [4.3-8.0]) (Figure 3a) in the severe RE group. No significant differences were observed in salivary pH (7.2 [7.0-7.4]/7.0 [6.7-7.2], p=0.1127) (Figure 3b) or acid-buffering capacity (male/female, 6.2 [5.7-6.5]/5.8 [5.3-6.2], p=0.1101) (Figure 3c) between males and females.

Sex differences in saliva secretion in the HC group

**Figure 4:**
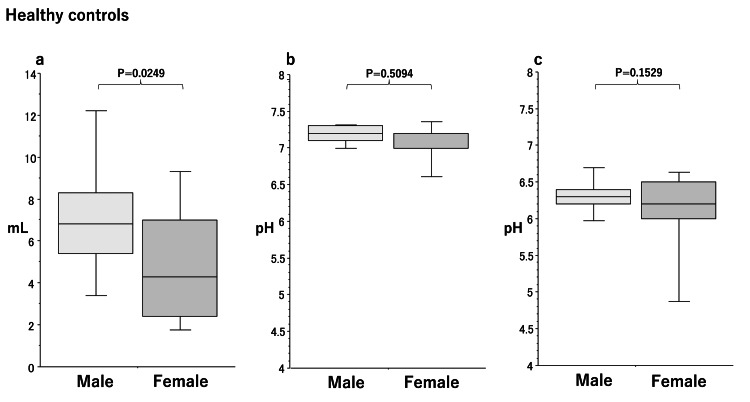
Box plots of sex differences in the amount of stimulated saliva secretion (a), salivary pH (b), and acid-buffering capacity (c) in healthy controls. Significant differences were calculated by the Mann-Whitney U test. Lower and upper whiskers indicate 10th and 90th percentiles, respectively. Box plots indicate 25th, 50th, and 75th percentiles

The amount of saliva secreted (Figure 4a) was significantly lower (p=0.0249) in females (4.3 [2.4-7.0]) than in males (6.8 [5.4-8.3]) in the HC group. No significant differences were noted in salivary pH (male/female, 7.2 [7.1-7.3]/7.2 [7.0-7.2], p=0.5094) (Figure 4b) or acid-buffering capacity (male/female, 6.3 [6.2-6.4]/6.2 [6.0-6.5], p=0.1529) (Figure 4c) between males and females.

Relationships between the amount of saliva secreted and height, body weight, and BMI

**Figure 5:**
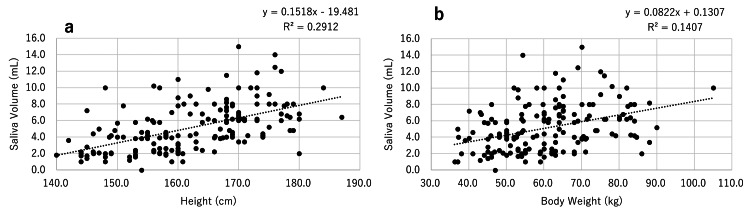
Relationships between the amount of saliva secreted and height (a) and body weight (b).

When the relationships between the amount of saliva secreted and height (Figure 5a), body weight (Figure 5b), and BMI were examined in all groups, the amount of saliva secreted positively correlated with height (p<0.001, R2=0.2912) and body weight (p<0.001, R2=0.1407); however, it did not correlate with BMI (p=0.193, R2=0.0095). A comparison of height and body weight revealed that the former showed a stronger correlation with the amount of saliva secreted.

## Discussion

The present study demonstrated that stimulated saliva secretion in GERD patients true NERD, mild RE, and severe RE) and HCs was significantly lower in females than in males. The results obtained revealed sex differences in saliva secretion in GERD patients and HCs. Since RE is caused by excessive esophageal acid exposure, and saliva secretion is associated with the duration of esophageal acid exposure, sex differences in saliva secretion may have an impact on the pathogenesis of GERD and, thus, warrant further study.

In the present study, the amount of saliva secreted by patients with mild RE was significantly lower in females, whereas salivary pH and acid-buffering capacity were similar in males and females. The cause of mild RE is excessive esophageal acid exposure with no difference in esophageal motility between patients with mild RE and HCs [23,24]; therefore, the secretion of a small amount of saliva may be a cause of excessive esophageal acid exposure in females with mild RE. A previous study compared the amount of saliva secreted between female patients with mild RE and female HHCs and showed that it was significantly lower in the former than in the latter [13]. Therefore, the lower amount of saliva secreted in female patients with mild RE was disease specific. We herein investigated sex differences in saliva secretion among patients with mild RE and the results obtained showed that the amount secreted was lower in females than in males. Although the amount of saliva secreted in female HCs was low, they did not have esophagitis; therefore, sex and RE may be attributed to a reduction in saliva secretion that results in excessive esophageal acid exposure. However, mild RE is more common in males, particularly younger males [1]. Multiple factors, including esophageal motility, saliva secretion, hiatus hernia, and being overweight, are associated with the onset of RE. Therefore, factors other than saliva secretion may have had an effect in male patients with mild RE.

The amount of saliva secreted by NERD patients was significantly lower in females, whereas salivary pH and acid-buffering capacity were similar in male and female RE patients. A previous study reported no significant differences in esophageal motility between NERD patients and HCs [25] and esophageal acid clearance was sufficiently maintained regardless of sex. The high incidence of NERD in females despite the absence of a sex difference in esophageal motility suggests that the secretion of a small amount of saliva plays a role in the development of symptoms in female NERD patients. In PCAB-resistant NERD patients who demonstrated a reflux-symptom association (a positive symptom index), the frequency of reflux symptoms was reported to be significantly higher when reflux fluid was pH 4-5 than when it was ≥pH 5 [26]. The pH of reflux fluid may be related to the development of reflux symptoms. Esophageal pH after acid reflux is lower in females than in males because the amount of saliva secreted is lower in females than in males. Therefore, reflux symptoms are more likely appear in females than in males. Moreover, the occurrence of reflux symptoms has been associated with a long duration of acid reflux and acid reflux to the upper esophagus [27]. In females, the secretion of a small amount of saliva may prolong the duration of acid reflux. Sex-dependent, disease-specific decreases in saliva secretion may also occur in patients with NERD; however, no studies to date have demonstrated a sex difference in saliva secretion in the NERD patient population. To further characterize differences in saliva secretion by sex, we intend to examine sex differences in NERD patients.

Male patients with severe RE were significantly younger than female patients. A previous study reported that more than 50% of the amount of stimulated saliva was secreted from the parotid gland and this was not affected by aging [28]. Therefore, in patients with severe RE, the effects of aging on the amount of saliva secreted are negligible. Regarding saliva secretion in patients with severe RE, the amount of saliva secreted was significantly lower in females, whereas salivary pH and acid-buffering capacity were similar in males and females. However, severe RE is also associated with hiatus hernia and impaired esophageal motility. These factors may also impact saliva secretion in patients with severe RE; however, the mechanisms by which saliva secretion independently affects the pathogenesis of severe RE have yet to be elucidated in detail.

Regarding the cause of low saliva secretion in females, a relationship with body size has been reported [15]. Therefore, we herein examined the relationships between saliva secretion and BMI, height, and body weight, and revealed correlations with both height and body weight, albeit more strongly with height. Based on these results, saliva secretion in females is considered to be related to body size, particularly height.

There are a number of limitations that need to be addressed. This was a single-center retrospective analysis of a small number of patients. Furthermore, we did not characterize differences in saliva secretion in GERD patients and HCs by sex. Moreover, female patients with severe RE were significantly older than males. The implementation of a prospective study on patients with similar characteristics is necessary in the future.

## Conclusions

We herein revealed a sex difference in saliva secretion in GERD patients, similar to HCs. The results obtained clearly showed that the amount of saliva secreted was significantly lower in female GERD patients than in male GERD patients. Further studies are warranted on sex differences in the amount of saliva secreted in the context of the pathophysiology of GERD.
